# All-van-der-Waals Heterostructure of MoS_2_ Grating and InSe Flake for Spectrally Selective Polarization-Sensitive Photodetection in NIR Region

**DOI:** 10.1021/acsnano.5c02102

**Published:** 2025-05-07

**Authors:** Yu-Te Chu, Po-Liang Chen, Shih-Hsiu Huang, Shyam Narayan Singh Yadav, Wei-Ren Syong, Ching-Han Mao, Yu-Jung Lu, Chang-Hua Liu, Pin Chieh Wu, Ta-Jen Yen

**Affiliations:** † Department of Materials Science and Engineering, 34881 National Tsing Hua University, Hsinchu 30013, Taiwan; ‡ Institute of Photonics Technology, 34881 National Tsing Hua University, Hsinchu 30013, Taiwan; § Department of Photonics, 34912 National Cheng Kung University, Tainan 70101, Taiwan; ∥ Research Center for Applied Sciences, 12381 Academia Sinica, Taipei 11529, Taiwan; ⊥ Department of Physics, National Taiwan University, Taipei 10617, Taiwan; # Center for Quantum Frontiers of Research & Technology (QFort), 34912 National Cheng Kung University, Tainan 70101, Taiwan; ∇ Meta-nanoPhotonics Center, 34912 National Cheng Kung University, Tainan 70101, Taiwan

**Keywords:** InSe, MoS_2_ gratings, van der Waals heterostructures, plasmonic enhancement, near-infrared, linear dichroism conversion, polarization-sensitive photodetectors

## Abstract

Near-infrared (NIR) polarization photodetectors based on 2D materials hold immense potential for numerous optoelectronic applications. To enhance the weak light-matter interaction in 2D materials, integrating 2D semiconductors with metallic plasmonic nanostructures presents an effective solution. However, such metallic plasmonic nanostructures suffer from high optical loss in the infrared region owing to inherent Ohmic losses in metals. By developing an all-van-der-Waals (vdW) heterostructure of MoS_2_ grating and InSe flake, we demonstrate a spectrally selective polarization-sensitive NIR photodetector. Herein, bulk MoS_2_ gratings possess lower optical loss and stronger field confinement compared to conventional metallic gratings, leading to a higher photoelectric conversion efficiency. Additionally, the MoS_2_ grating supports both TE-excited guided-mode resonance at λ = 790 nm and TM-excited plasmonic resonance at λ = 960 nm. Such linear dichroism conversion behavior, with wavelength tunability, enables spectrally selective polarization-sensitive photodetection in the NIR region, achieving high dichroic ratios of 1.61 at 790 nm and 1.88 at 960 nm. Under 960 nm illumination, such MoS_2_ grating/InSe flake photodetector also demonstrates a responsivity of 28.5 A/W and a detectivity of 9.81 × 10^12^ Jones, respectively. In addition, with an ultrafast rise time of 195 ns and a decay time of 222 ns, this device represents the fastest photoresponse speed among InSe-based photodetectors reported to date. These results highlight the potential of 2D semiconductor gratings for high-performance all-vdW optoelectronics and nanophotonic devices.

## Graphical abstract

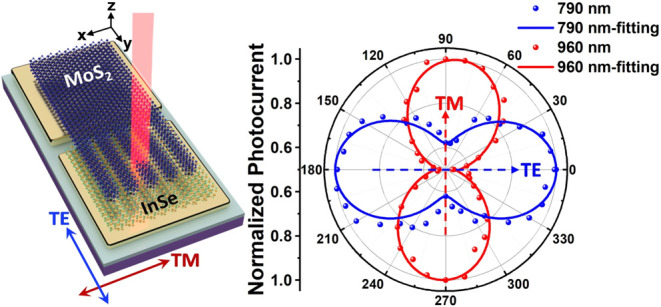

## Introduction

Near-infrared (NIR) photodetectors play a crucial role in numerous optoelectronic applications, including thermal imaging, optical communication, environmental monitoring, and biological sensing.^1–4^ In recent decades, NIR polarization photodetectors have garnered further significant attention because of their substantial potential in remote sensing, optical navigation, and quantum computing.^5–7^ In particular, spectrally selective polarization-sensitive photodetector for NIR light, capable of detecting two orthogonal TE- and TM-polarized waves at different wavelengths, appears highly desirable for advanced applications such as polarization-sensitive optical coherence tomography (PS-OCT) imaging and light detection and ranging (LiDAR).^8–10^

To develop a novel NIR photodetector, employing two-dimensional (2D) layered materials appears highly promising due to their unique properties, including the ability of shrinking the size of photodetectors, comprehensive coverage of the bandgap, and easy integration to construct vdW heterostructures without the issue of lattice mismatch.^11,12^ Among 2D materials, the metal monochalcogenide III–VI compound, Indium selenide (InSe), has attracted significant attention owing to its outstanding electrical and optoelectronic properties beyond other 2D materials for NIR photodetection, such as BP, MoS_2_, MoTe_2_, etc.^13–15^ For example, InSe possesses a smaller electron effective mass (m* = 0.143 m_0_) compared to MoS_2_ (m* = 0.45 m_0_), and exhibits high carrier mobility over 10^3^ cm^2^ V^–1^ s ^–1^ at room temperature.^16,17^ InSe also possesses much higher chemical stability compared to 2D black phosphorus (BP), which suffers from inherent air instability for practical applications.^5^ Furthermore, unlike commonly used multilayer transition-metal dichalcogenides (TMDC), which present an indirect bandgap, multilayer InSe exhibits a layer-independent narrow direct bandgap (E_g_ = 1.26 eV).^18,19^ These aforementioned advantages render InSe an excellent material for NIR light detection. However, the intrinsic challenges with 2D InSe lie in its low absorption and small optical cross-section due to its intrinsically atomically thin nature, which results in reduced photoelectric conversion efficiency in most 2D-based optoelectronic devices.^20,21^ Besides, most reported InSe-based photodetectors only demonstrate high photoresponsivity in the ultraviolet (UV) to visible (VIS) wavelength regions, whereas their performance in the NIR region remains limited.^16,22,23^

To enhance the weak light-matter interaction in InSe, integrating 2D semiconductors with plasmonic nanostructures has proven to be an effective strategy.^24,25^ Plasmonic nanostructures enable excitation of surface plasmon resonance (SPR) and localized SPR (LSPR), which can significantly confine and enhance the local electromagnetic (EM) fields within 2D materials, resulting in higher quantum efficiency and photoresponsivity. Based on this mechanism, several metal plasmonic nanostructures (e.g., Au, Ag, Al) have been proposed to boost the optoelectronic performance of photodetectors.^20,26–29^ Nevertheless, metals typically exhibit a greater extinction coefficients (k), which increase with longer wavelengths, leading to high optical losses in the NIR region.^30^ As a result, metallic plasmonic nanostructures exhibit low-quality resonances owing to inherent Ohmic losses in metals.^31^ In contrast, bulk TMDC materials (e.g., MoS_2_, WS_2_) possess a nearly zero extinction coefficient in the NIR region and a high refractive index (*n* > 4) across the VIS to NIR regions, rendering them suitable for constructing subwavelength optical resonators.^32,33^ Such TMDC nanoresonators, with their inherent low loss compared to metals, can excite resonances with high quality-factor (Q-factor), leading to longer propagation distances of surface plasmon polaritons (SPPs).^31,34^ Moreover, bulk TMDC exhibits a refractive index in the NIR region that is over 20% higher than that of silicon, a classical material for all-dielectric metasurfaces and silicon photonics, indicating stronger optical field confinement and a smaller effective mode area for TMDC waveguides.^35^ Based on the aforementioned advantages, the optical properties of TMDC nanophotonics have attracted significant research interest, while their optoelectronic properties remain underexplored.

In this work, we proposed an all-vdW photodetector, composed of MoS_2_ grating and InSe flake, achieving high-performance spectrally selective polarization-sensitive photodetection in the NIR region. To enhance the light-matter interaction of InSe in the NIR region, we applied multilayer MoS_2_ gratings with low k and high n values instead of conventional metallic gratings, which typically suffer from a high optical loss in the infrared region. Such a MoS_2_ grating works as a multifunctional structure as follows: (1) Bulk MoS_2_ is also a NIR photoactive material with an indirect bandgap of 1.23 eV. (2) Bulk MoS_2_ forms type-II band alignment with InSe, contributing to a longer carrier lifetime due to the charge separation of photogenerated carriers driven by the built-in electric field at the interface. (3) MoS_2_ gratings function as high Q-factor resonators with low optical loss and strong field confinement. Both simulated and measured polarization-dependent spectra demonstrate that the MoS_2_ grating can support not only plasmonic resonance at the MoS_2_ grating/Au interface under transverse magnetic (TM) polarization at λ = 960 nm, but also guided-mode resonance within the MoS_2_ grating under transverse electric (TE) polarization at λ = 790 nm, leading to a linear dichroism conversion phenomenon. Linear dichroism (LD) conversion, characterized by an orthogonal reversal of polarization-sensitive absorption signals and photocurrent, typically arises from the intrinsic structural anisotropy of materials, which limits the modulation of their operating wavelength.^36–39^ In contrast, the LD conversion property in our device, driven by polarization-dependent excitation of different resonance modes, offers flexible wavelength tunability, enabling spectrally selective polarization-sensitive photodetection with high dichroic ratios of 1.61 at 790 nm and 1.88 at 960 nm. Furthermore, our device exhibits an enhanced responsivity of 28.5 A/W and a detectivity of 9.81 × 10^12^ Jones under 960 nm laser illumination, with an ultrafast rise (195 ns) and decay (222 ns) times. These findings suggest that TMDC gratings serve as effective alternatives to conventional metallic plasmonic structures for subwavelength nanophotonic applications.

## Results and Discussion

To fabricate the MoS_2_ grating/InSe flake polarization-sensitive photodetector, the top-down fabrication process was adopted, as shown in Figure 1a. First, Au/Cr electrodes were fabricated on SiO_2_/P^+^-Si substrates (Step I). Subsequently, mechanically exfoliated multilayer InSe and MoS_2_ flakes were vertically stacked on one electrode via the traditional dry transfer technique^40^ to form MoS_2_/InSe heterojunction (Steps II and III). To pattern the MoS_2_ grating, PMMA was coated on the surface of the device, and the vertically stacked MoS_2_/InSe region was patterned using electron-beam lithography (EBL) (Steps IV and V). Then, the exposed MoS_2_ was removed by inductively coupled plasma reactive-ion etching (ICP-RIE) without damaging the InSe beneath due to the high etching selectivity (Step VI). Finally, the PMMA coating is stripped to obtain the designed MoS_2_ grating/InSe flake photodetector (Step VII). Detailed information on the fabrication process can be found in the Methods.

**Figure 1:**
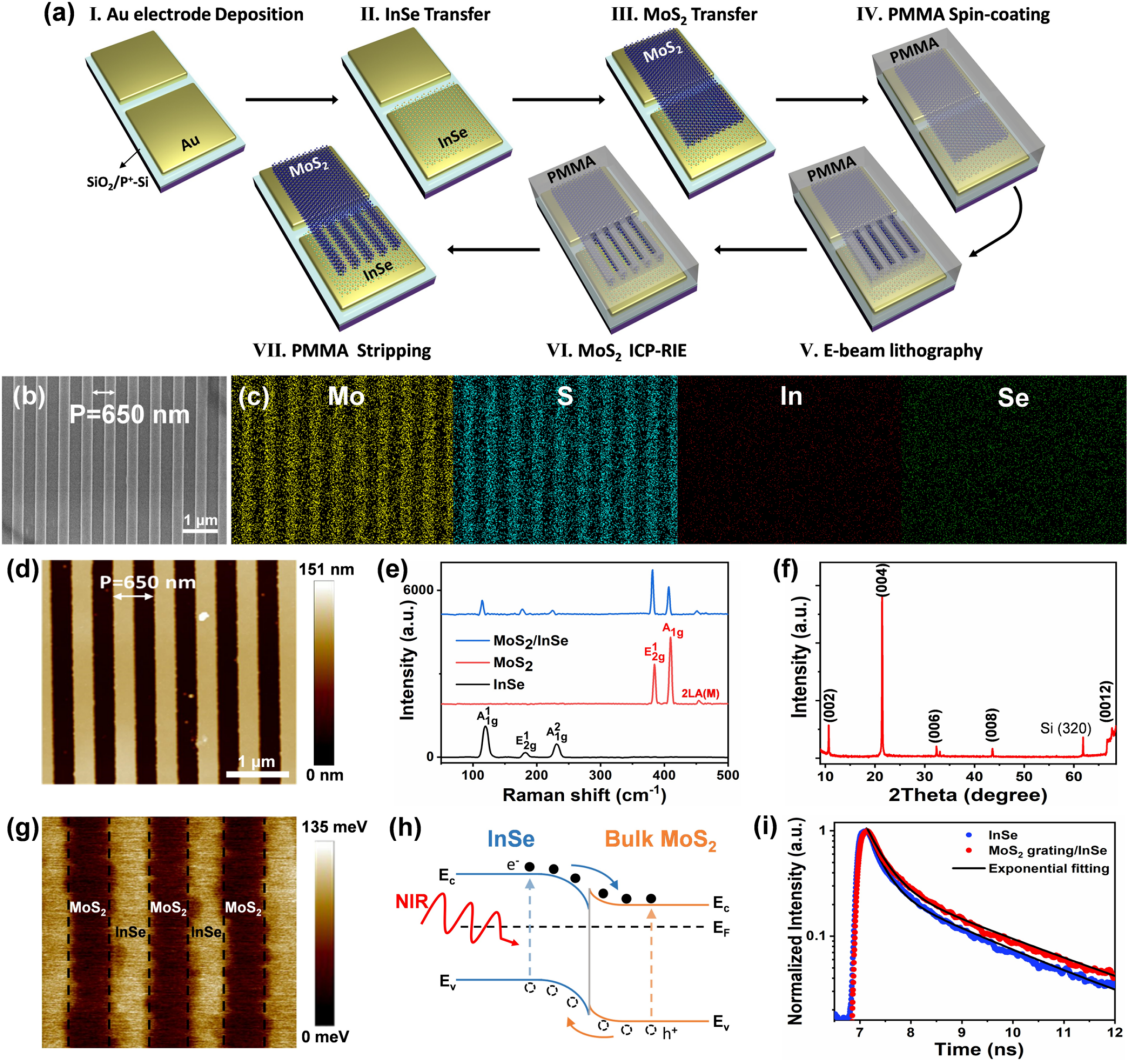
Device structure and characterization of MoS_2_ grating/InSe flake photodetector. (a) Schematic diagram of the fabrication process for MoS_2_ grating/InSe flake photodetector. (b) SEM image of MoS_2_ grating/InSe flake heterostructure and (c) the corresponding EDS mapping of Mo, S, In, and Se elements. (d) AFM height image of MoS_2_ grating/InSe flake heterostructure. (e) Raman spectra taken from InSe, MoS_2_, and MoS_2_ grating/InSe flake heterojunction, respectively. (f) XRD pattern of InSe flakes on SiO_2_/Si substrate. (g) Work function difference (ΔΦ) mapping image of MoS_2_ grating/InSe flake heterostructure. (h) Schematic energy-band diagram of MoS_2_ grating/InSe flake heterojunction without applied bias (V_ds_ = 0 V). (i) Normalized TRPL spectra of the pristine InSe and MoS_2_ grating/InSe flake heterostructure. The exponential fitting curves of both trends are shown with black solid line.

The morphologies of the as-fabricated MoS_2_ grating/InSe flake heterostructure were characterized by optical microscopy (OM), scanning electron microscopy (SEM), and atomic force microscopy (AFM). The OM images of the MoS_2_ grating/InSe flake photodetector are shown in Figure S1a–d. The dark regions in the bright-field OM image (Figure S1a) and the bright regions in the dark-field OM image (Figure S1b) both correspond to the MoS_2_ grating, indicating its high extinction properties. As shown in Figure 1b, the SEM image demonstrates that the patterned MoS_2_ grating structure is well-defined, with a grating period of 650 nm and a filling ratio of approximately 0.5. In addition, the atomic force microscopy (AFM) images of the MoS_2_ grating/InSe flake also revealed identical dimensional parameters (Figure 1d). Figure S2 presents the corresponding height profiles, which indicate that the height of the MoS_2_ grating is 136 nm and the thickness of the InSe flake is 36 nm. In summary, AFM mapping shows a smooth surface with excellent uniformity, which is crucial for minimizing unwanted scattering and achieving higher efficiency in an optical resonator.^41^

In addition to scrutinizing the morphologies of the as-fabricated MoS_2_ grating/InSe flake heterostructure, we further examined its chemical compositions and crystal structure. For example, energy dispersive X-ray spectroscopy (EDX) mapping over the MoS_2_ grating/InSe flake heterostructure and the pure InSe flakes confirm that such a heterostructure is composed of high-quality, impurity-free MoS_2_ and InSe, as shown in Figures 1c and S3, respectively. The crystal structure of the MoS_2_ grating/InSe flake heterojunction was also characterized by Raman spectroscopy and X-ray diffraction (XRD). As displayed in Figure 1e, the Raman spectrum of InSe shows three peaks at 119, 182, and 231 cm^–1^, which are attributed to A_1g_^1^, E_2g_^1^, and A_1g_^2^, respectively.^16^ The phonon modes of MoS_2_, E_2g_^1^ at 385 cm^–1^, A_1g_ at 410 cm^–1^, and 2LA­(M) at 454 cm^–1^, can also be identified.^42^ In the heterojunction region, the Raman spectrum exhibits prominent modes of both InSe and MoS_2_, indicating the existence of two distinct materials. Besides, as shown in Figure 1f, the XRD pattern exhibits five distinct peaks at 10.7, 21.4, 32.4, 43.6, and 67.7°, corresponding to the (002), (004), (006), (008), and (0012) planes of the InSe crystal, respectively (PDF34–1431). Based on the Raman spectra and XRD results, the InSe flakes are confirmed to be β-InSe, belonging to the *P*6_3_/*mmc* centrosymmetric space group, which is polarization-insensitive.^12^ However, polarization sensitivity can be achieved by integrating β-InSe with an anisotropic MoS_2_ grating.

Furthermore, to investigate the energy band alignment between MoS_2_ and InSe, the work function difference mapping was measured by Kelvin probe force microscopy (KPFM), as shown in Figure 1g. The measured work function difference ΔΦ_InSe_–Φ_MoS2_ is 63.7 meV. Such a work function difference will lead to a large built-in potential after Fermi level alignment upon contact. The corresponding type-II band alignment diagram of the MoS_2_/InSe heterostructure is presented in Figure 1h.^43^ Driven by the built-in potential at the interface, photogenerated electrons and holes easily transfer across the junction into MoS_2_ and InSe, respectively, resulting in a decreased recombination rate and a longer carrier lifetime. The carrier dynamics in the device were further characterized by time-resolved photoluminescence (TRPL), as shown in Figure 1i. The TRPL curves exhibit multiexponential decays and can be fitted by a biexponential decay function *Ae* ^–*t*/τ1^ + *Be* ^–*t*/τ2^, where τ_1_ < τ_2._ ^26^ τ_1_ is attributed to the carrier nonradiative recombination at the interface, whereas τ_2_ is associated with the carrier radiative recombination that mainly occurs within the bulk crystals.^44^ The resulting decay time constants for pristine InSe are 0.27 ns (τ_1_) and 2.04 ns (τ_2_), while the MoS_2_ grating integrated InSe shows decay time constants of 0.33 ns (τ_1_) and 2.33 ns (τ_2_), which are 22% and 14% longer, respectively. The prolonged carrier lifetime in MoS_2_ grating/InSe flake heterostructure may be attributed not only to the built-in potential at the interface but also to the strong surface plasmon-exciton interaction.^45–47^

To investigate the resonance mode of the MoS_2_ grating prior to its integration with InSe, we performed a series of numerical simulations using COMSOL Multiphysics. These simulations calculated the reflectance (R), transmittance (T), and the corresponding electromagnetic field distributions. The schematic of the designed MoS_2_ grating on an Au mirror is shown in Figure 2a. Here, P, W, and H denoted the period, width, and height of the MoS_2_ grating, respectively. The filling ratio, defined as W/P, is fixed at 0.5, and the height is set as 136 nm based on the AFM results shown in Figure S2a. The multilayer MoS_2_ grating, with a high refractive index (*n* > 4) across the VIS to NIR regions,^48^ acts as an optical resonator, while the Au mirror functions as both a conductive electrode and a metallic bottom layer that supports surface plasmon resonance. To design the MoS_2_ grating with resonance in the NIR region, a parameter sweep of the grating period and wavelength was conducted under the normal incidence of TM-polarized light. As shown in Figure 2b, the simulated absorption spectra as a function of periods reveal dual perfect absorption bands in the NIR region. The corresponding magnetic field distributions are labeled with matching symbols in Figure 2c. The magnetic resonance (cross symbol), driven by circulating currents, is generated within the MoS_2_ grating, while the third-order surface plasmon resonance (ring symbol) is excited at the interface between the MoS_2_ grating (ε > 0) and the Au substrate (ε < 0). Arising from the additional wavevector provided by the MoS_2_ grating, the resonance wavelength of the third-order SPR mode is highly dependent on the grating period (Figure 2b) but nearly independent of the grating height (Figure S4a). As a result, the resonance wavelength of such a SPR mode can be precisely tuned by adjusting the grating period, in contrast to the magnetic resonance mode, which is sensitive to random variations in grating height (Figure S4). By modulating the resonance wavelength of the third-order SPR mode, we determined the optimal geometrical parameters to be P = 650 nm, W = 325 nm, and H = 136 nm, which are consistent with the structural parameters of the as-fabricated sample, as confirmed by SEM and AFM measurements (Figure 1b,g).

**Figure 2:**
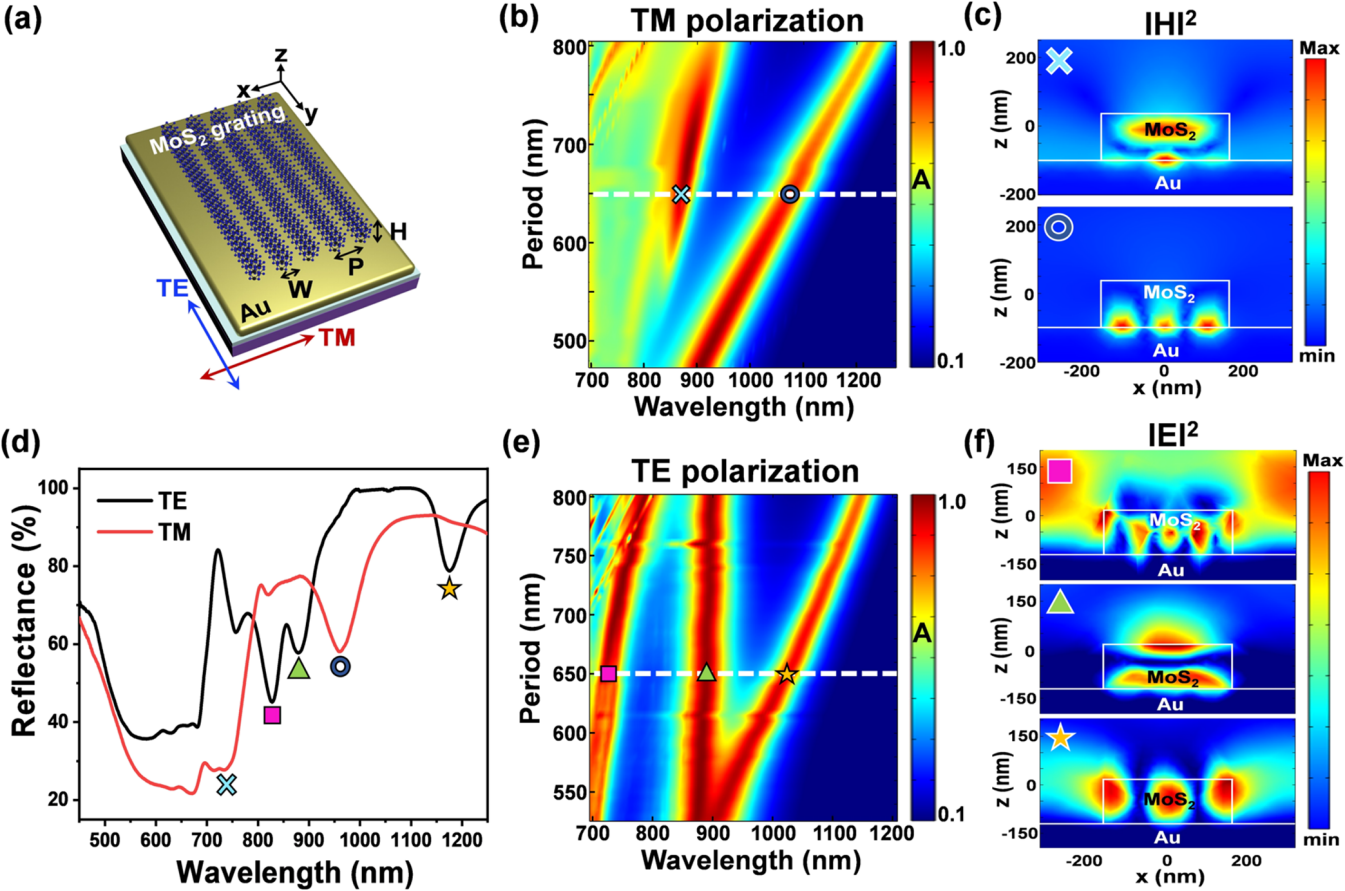
Resonance absorption in MoS_2_ grating on Au substrate under TM and TE polarizations. (a) Schematic illustration of MoS_2_ grating on Au substrate. (b) Color map of COMSOL simulated absorption spectra under TM polarization as a function of the grating period with a fixed filling ratio of 0.5 and height of 136 nm. The absorption of the structure is calculated using the equation A = 1 – R – T, where *T* = 0 owing to the suppression of transmittance by the 100 nm-thick Au bottom layer, which exceeds the penetration depth of the electromagnetic wave. (c) Magnetic field distributions of the optimized structure (*P* = 650 nm) for the magnetic resonance mode (cross symbol) and the third-order SPR mode (ring symbol), as marked with corresponding symbols in panel (b). (d) Experimentally measured reflectance spectra of the designed MoS_2_ grating under TE and TM polarizations. The peaks corresponding to various resonance modes are labeled with matching symbols in panels (c, f). (e) Color map of COMSOL simulated absorption spectra as a function of the grating period under TE polarization. (f) Electric field distributions of the multiple guided-mode resonances: TE_51_ (square symbol), TE_12_ (triangle symbol), and TE_31_ (star symbol) modes are marked with corresponding symbols in (e).

Subsequently, we conducted experimental measurements of reflectance spectra for the as-fabricated MoS_2_ grating on an Au substrate under TM- and TE-polarized illuminations, as shown in Figure 2d. Under TM polarization, two reflectance valleys (i.e., absorption peaks) were observed, corresponding to the magnetic resonance at λ = 733 nm (cross symbol) and the third-order surface plasmon resonance at λ = 960 nm (ring symbol), near the band edge of bulk InSe (λ = 984 nm), as predicted by the simulations. The slight wavelength shift between the experimental and simulated spectra may arise from differences in the optical constants of the materials between the database and actual samples. On the other hand, under TE polarization, three reflectance valleys were observed at wavelengths of λ = 828, 880, and 1175 nm, corresponding to multiple guided-mode resonances: TE_51_ (square symbol), TE_12_ (triangle symbol), and TE_31_ (star symbol), respectively. These guided-mode resonances were further confirmed by the simulated absorption spectra (Figure 2e) and the corresponding electric field distributions, labeled with matching symbols, are shown in Figure 2f. The guided-mode resonances exhibit strong cavity size dependence, allowing their resonance wavelengths to be tuned by adjusting both the grating period (Figure 2e) and the grating height (Figure S4b).

To further reveal the distinctive optical response of this 2D-based MoS_2_ grating compared to that of conventional metallic gratings, an Ag grating with the same geometrical parameters as the MoS_2_ grating was fabricated, and their reflectance spectra were measured for comparison under TM and TE polarization, as shown in Figure S5a,b, respectively. Overall, the MoS_2_ grating not only exhibits a 229% higher quality factor (Q-factor) for the SPR mode than the Ag grating under TM polarization (Figure S5a), indicating the inherent low-loss properties of the MoS_2_ grating, but also demonstrates additional guided-mode resonances under TE polarization (Figure S5b), which do not occur in conventional metallic gratings.

Afterward, we transferred an InSe flake to the interface between the MoS_2_ grating and the Au electrode, as illustrated in Figure 3a, and then explored the optical properties of the MoS_2_ grating/InSe flake heterostructure. First, we investigated the polarization-dependent resonance behaviors by measuring the polarization-dependent reflectance spectra of the MoS_2_ grating/InSe flake photodetector, as shown in Figure 3b. As the polarization angle changes from 0° (TE) to 90° (TM), the reflectance gradually increases at λ = 790 nm, whereas it decreases at λ = 960 nm. Additionally, the polarization-dependent absorption spectra, extracted from Figure 3b, indicates that the absorption at λ = 790 nm reaches a maximum under TE polarization, while the absorption at λ = 960 nm reaches a maximum under TM polarization, as shown in Figure 3c. Such an LD conversion phenomenon originates from the TE-excited TE_51_ guided-mode resonance at λ = 790 nm and the TM-excited hybrid surface plasmon resonance/magnetic resonance mode at λ = 960 nm, thereby enabling our device to realize spectrally selective polarization-sensitive photodetection. Notably, these resonance modes provide LD conversion property with superior wavelength tunability compared to other reported systems based on inherent structural anisotropy of materials.^36,37,49^ Next, the corresponding electromagnetic field distributions of these two resonance bands are displayed in Figure 3d,e. Both figures reveal that the resonance hotspots overlap with the InSe flake at the interface between the MoS_2_ grating and Au, which thus significantly increases the light-matter interaction of InSe owing to the strong local field enhancement.

**Figure 3:**
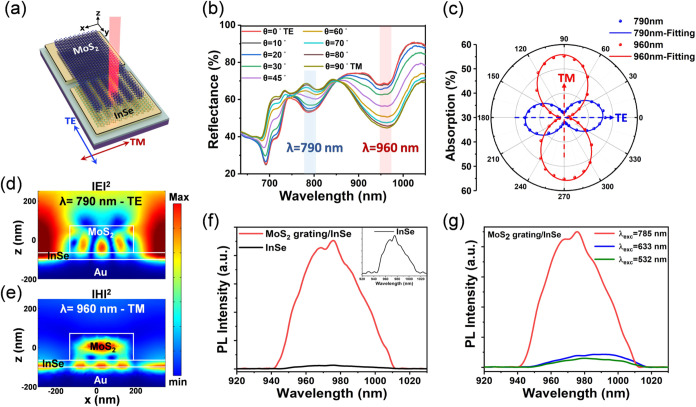
Optical properties of the MoS_2_ grating/InSe flake heterostructure. (a) Schematic of the MoS_2_ grating/InSe flake photodetector. (b) Measured polarization-dependent reflectance spectra of MoS_2_ grating/InSe flake photodetector, with the polarization angle θ ranging from 0° (TE) to 90° (TM) in 10 steps. (c) Polar plot of the absorption of MoS_2_ grating/InSe at λ = 790 and 960 nm as a function of linear polarization angle of incident light. (d) Electric field distribution and local field enhancement for MoS_2_ grating/InSe at λ = 790 nm under TE polarization. (e) Magnetic field distribution and local field enhancement for MoS_2_ grating/InSe at λ = 960 nm under TM polarization. (f) Steady-state photoluminescence spectra for MoS_2_ grating/InSe and bare InSe, measured with 785 nm excitation wavelength at 10 mW power. The upper inset shows the zoom-in PL spectrum of bare InSe. (g) Steady-state photoluminescence spectra for MoS_2_ grating/InSe, measured with 532 (off-resonance), 633 (off-resonance), and 785 nm (on-resonance) excitation wavelengths, each at a power of 10 mW.

Such enhancement of the light-matter interaction is directly evaluated by conducting photoluminescence (PL) spectra measurements. As shown in Figure 3f, PL spectra of MoS_2_ grating/InSe and bare InSe were measured with an excitation wavelength of 785 nm, which matches well with the aforementioned guided-mode resonance of MoS_2_ grating/InSe at λ = 790 nm. The MoS_2_ grating/InSe sample exhibited a 35-fold enhancement in PL intensity compared to bare InSe, with a PL peak position at around 975 nm. Furthermore, PL spectra of MoS_2_ grating/InSe were measured with different excitation wavelength of 532 nm (off-resonance), 633 nm (off-resonance), and 785 nm (on-resonance), as shown in Figure 3g. Despite the higher intrinsic absorption at 532 and 633 nm as compared with that at 785 nm in the MoS_2_ grating/InSe, it is observed that only excitation at 785 nm presents pronounced enhancement, proving that the significantly enhanced PL intensity is attributed to the presence of guided-mode resonance from the MoS_2_ grating.

Subsequently, we scrutinized the optoelectronic characteristics of the MoS_2_ grating/InSe flake photodetector by measuring its I–V responses at resonance wavelengths. Figure 4a,4b shows the I_ds_-V_ds_ curves of the MoS_2_ grating/InSe flake photodetector at V_ds_ from −2 to 0.5 V with MoS_2_ grounded, under different power densities of both 790 and 960 nm laser illumination, respectively. Note that our device is measured without applying any back-gate voltage, which enhances its energy efficiency and thus commit it more feasible for practical applications. At both wavelengths, the rectifying I_ds_-V_ds_ curves correlate with the type-II band alignment formed at the MoS_2_/InSe heterointerface, which is consistent to the band alignment diagram presented in Figure 1h. Under illumination, photogenerated carriers could be dissociated by the built-in electric field to generate photocurrent without applying external drain bias, resulting in a slightly photovoltaic behavior (Figure 4a,b). Moreover, under a reverse bias of −2 V, the photocurrent (I_ph_ = I_light_ – I_dark_) increases dramatically with the illumination light power density, as shown in Figure 4c. The fitting curve (I_ph_ ∝ P^α^) exhibit a near-linear relationship (α ≈ 0.93) between photocurrent and illumination power density at λ = 960 nm, indicating that the dominant mechanism should be the photoconductive effect.^50^ On the other hand, a nonlinear relationship (α *≈* 0.77) is observed at λ = 790 nm, suggesting a complex process involving carrier generation, trapping, and recombination in our device.^51^

**Figure 4:**
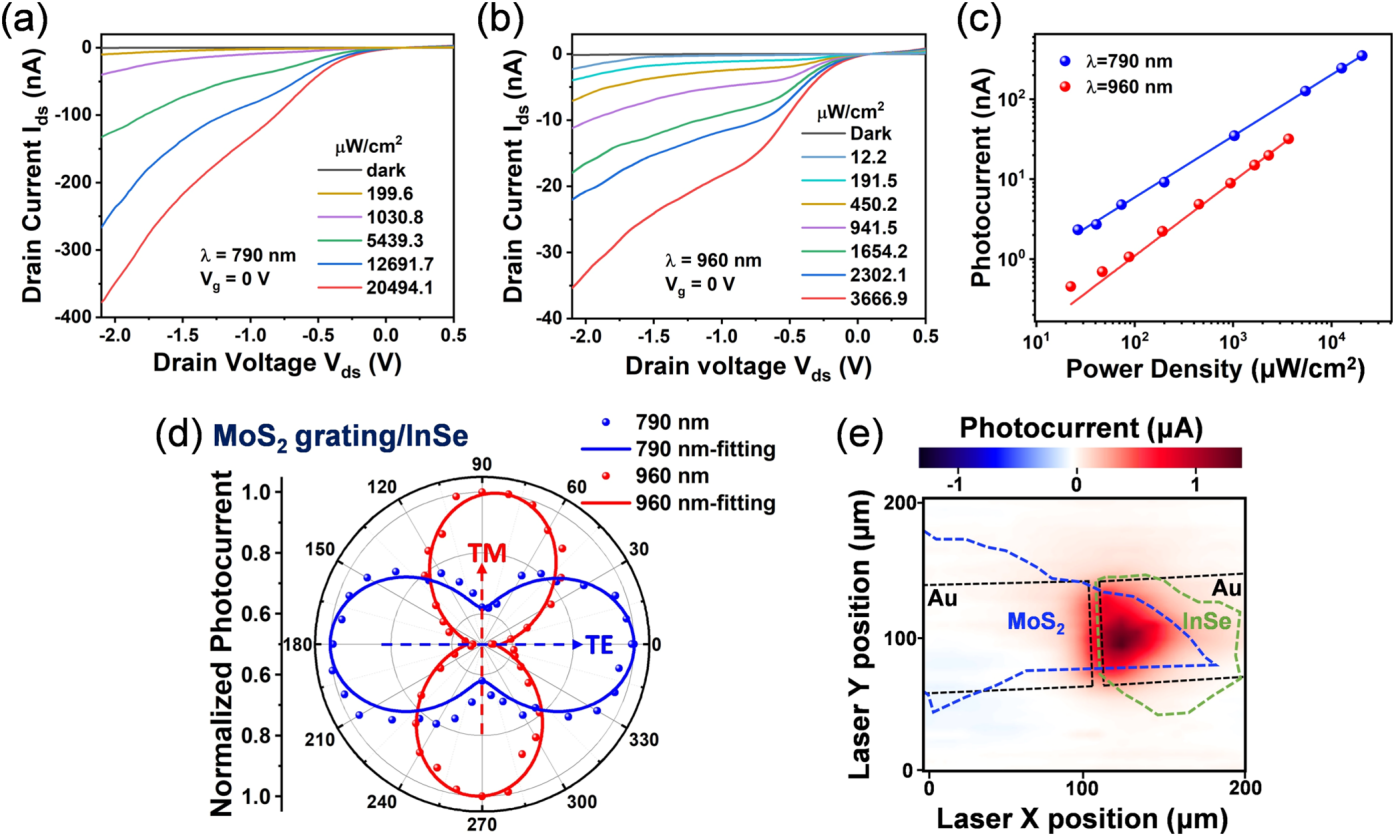
Optoelectronic characterization of the MoS_2_ grating/InSe flake photodetector. I–V characteristics of the MoS_2_ grating/InSe flake photodetector under illumination of (a) 790 nm and (b) 960 nm laser with various power densities at V_g_ = 0 V. (c) Illumination power dependence of photocurrent under 790 and 960 nm laser at V_ds_ = −2 V. (d) Polarization-dependent normalized photocurrent for MoS_2_ grating/InSe device at λ = 790 and 960 nm. (e) Spatially resolved two-dimensional photocurrent map (illumination wavelength: 785 nm).

Next, we further probed the polarization-dependent photocurrent to verify the polarization sensitivity of the MoS_2_ grating/InSe flake photodetector, as presented in Figure 4d. It shows that our device exhibits sensitivity to TE-polarized light at λ = 790 nm and TM-polarized light at λ = 960 nm, which agree well with the polarization-dependent absorption results illustrated in Figure 3c. Such orthogonal reversal of photocurrent at different wavelengths is attributed to the LD conversion, which enables spectrally selective polarization-sensitive photodetection.^36–38^ Moreover, our device exhibits excellent dichroic ratios (I_pmax_/I_pmin_) of 1.61 at 790 nm and 1.88 at 960 nm, which are higher than those of many reported 2D anisotropic materials and heterostructures, such as GeSe (1.09 at 532 nm),^52^ ReS_2_/ReSe_2_ (1.2 at 638 nm),^53^ and GeAs (1.49 at 520 nm).^36^ In contrast, the photocurrent of the planar MoS_2_/InSe device remains unchanged with different polarization angles, indicating that the device shows no polarization sensitivity without the anisotropic grating structure (Figure S6). Consequently, it can be concluded that the polarization-sensitive response of the MoS_2_ grating/InSe flake photodetector is induced by the TE-excited guided-mode resonance at λ = 790 nm and TM-excited surface plasmon resonance at λ = 960 nm, supported by the patterned MoS_2_ grating.

Furthermore, we conducted scanning photocurrent microscopy^54,55^ to spatially resolve the photoresponses of the MoS_2_ grating/InSe flake photodetector. Figure 4e displays the scanning photocurrent image obtained by scanning a focused laser beam (λ = 785 nm) across the entire photonic structure under a bias of −2 V. The scanning photocurrent image reveals a single hot spot corresponding to illumination on the MoS_2_ grating/InSe flake heterostructure. Such a result further manifests that both enhanced photocurrent and polarization sensitivity indeed originate from the MoS_2_ grating/InSe flake heterostructure.

To reveal the superior photodetection performance of the MoS_2_ grating/InSe flake photodetector, here, we analyzed two critical figures of merit (FOM), responsivity (R) and detectivity (D*). Responsivity (R) quantifies the efficiency of photocurrent generation in a photodetector, and it can be expressed as below,

1 $$R_{\lambda }=\frac{\left|I_{\mathrm{ph}}\right|}{P}$$

where I_ph_ denotes the photocurrent (I_ph_ = I_light_ – I_dark_), P the illumination power, and λ the excitation wavelength. Notably, R typically decreases with increasing illumination power, owing to the saturation of light absorption and the increased photocarrier recombination rate in the photodetector. We evaluated the power-dependent photoresponsivity under 790 and 960 nm laser illumination at V_ds_ = −2 V and V_g_ = 0 V for four types of devices: MoS_2_ grating/InSe, Ag grating/InSe, MoS_2_/InSe, and pristine InSe, as shown in Figure 5a,b. Among those four devices, the MoS_2_ grating/InSe device exhibits the highest photoresponsivity, with a maximum R of 17.6 A/W under a 790 nm laser and 28.5 A/W under a 960 nm laser, showing significant enhancements of 152- and 721-fold compared to the pristine InSe device, respectively. It is worth noting that the MoS_2_ grating/InSe device also possesses a 13-fold higher responsivity than the Ag grating/InSe device under 960 nm laser, evidently indicating higher enhancement factors and external quantum efficiency (EQE) due to the inherent lower loss of MoS_2_ grating.

**Figure 5:**
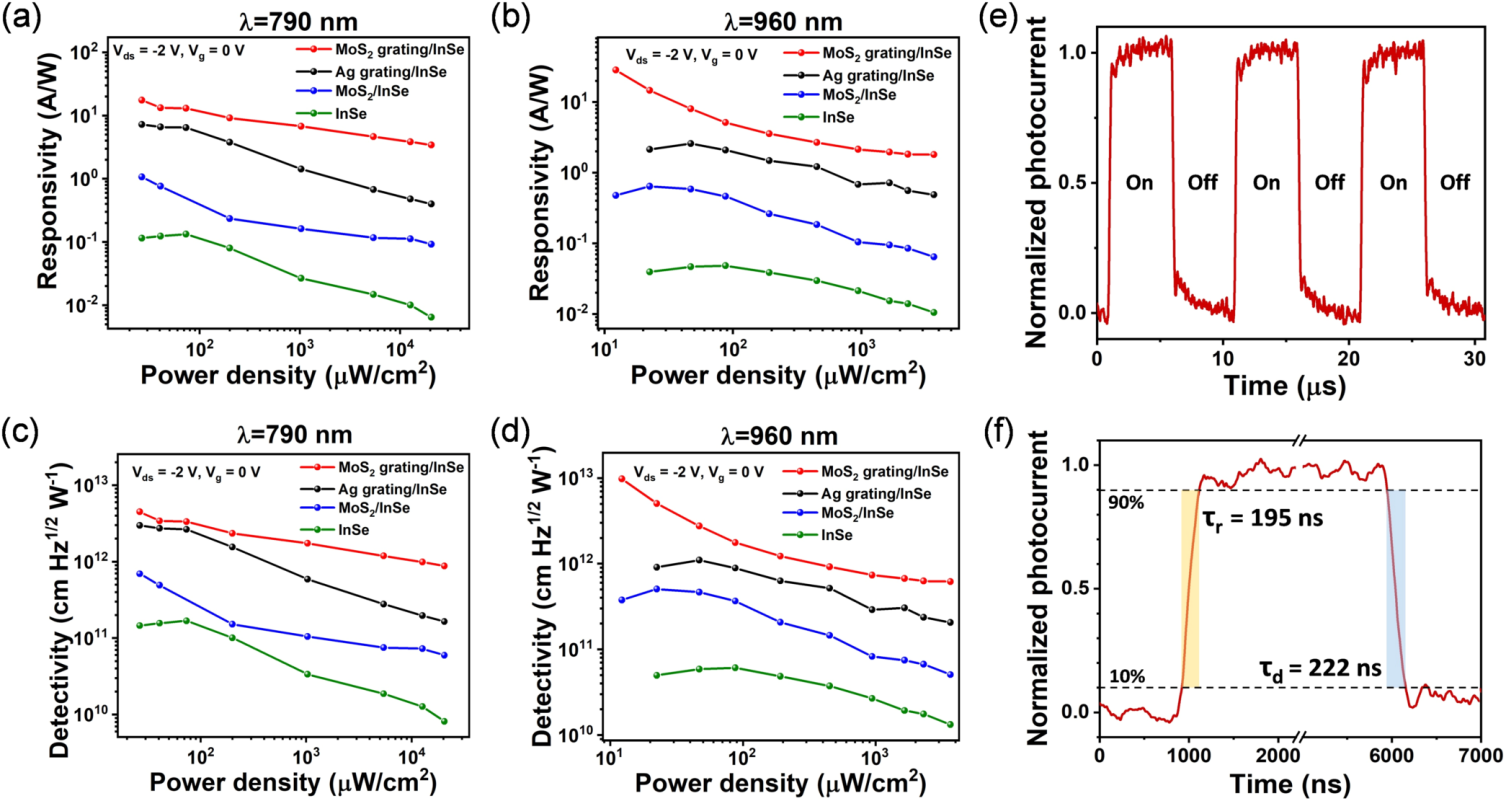
(a, b) Calculated photoresponsivity (R) and (c, d) detectivity (D*) versus illumination power density for four types of photodetector devices under 790 and 960 nm laser illumination, respectively. (V_ds_ = −2 V and V_g_ = 0 V). (e) Time-resolved photoresponse of MoS_2_ grating/InSe device at fixed V_ds_ = −2 V under 785 nm laser illumination modulated at a frequency of 100 kHz. (f) Analysis of the 10–90% rise time (τ_r_) and 90–10% decay time (τ_d_).

Besides, the other evaluated FOM for the photodetectors is detectivity (D*), which describes the weakest detectable signal. As shot noise dominates the total noise, detectivity (D*) can be expressed as below,

2 $$D^{*}=\frac{\sqrt{\mathrm{AB}}}{\mathrm{NEP}}\approx \frac{R_{\lambda }}{\sqrt{\frac{2qI_{d}}{A}}}$$

where A is active area of device (∼500 μm^2^), B is the bandwidth, q is the elementary charge, I_d_ is the dark current, and R_λ_ is the responsivity. In Figure 5c,d, the calculated D* exhibits the same enhanced trend as that of responsivity, reaching a maximum D* of 4.51 × 10^12^ Jones under 790 nm laser and 9.81 × 10^12^ Jones under 960 nm laser for the MoS_2_ grating/InSe device. The D* values obtained are notably high compared to those of other InSe-based photodetectors in the NIR region.^11,18,56,57^ Such ultrahigh detectivity arises not only from the MoS_2_ grating enhanced responsivity but also from the low value of dark current (131 pA) suppressed by the large Schottky barrier height between InSe and the Au contact.

Finally, we measured the response speed of the MoS_2_ grating/InSe flake photodetector, which determines the ability of the photodetector to track fast-switching optical signals. Figure 5e shows the time-resolved photoresponse of the MoS_2_ grating/InSe device under periodic on/off illumination of a 785 nm laser modulated at 100 kHz using an acousto-optic modulator (AOM). Notably, the photoresponse remains steady and repeatable across cycles of on/off illumination, demonstrating the excellent stability and reproducibility of our device. Furthermore, to examine the modulation speed, the transient photocurrent responses were analyzed, as shown in Figure 5f. The resolved 10–90% rise time (τ_r_) and 90–10% decay time (τ_d_) are 195 and 222 ns, respectively, representing the fastest photoresponse speed reported to date for InSe-based photodetectors. This ultrafast response speed may arise from the strong built-in electric field and the effective exciton–plasmon coupling process.^16,58–60^ To summarize, we also compared the optoelectronic performance of our device with other reported InSe-based NIR photodetectors, as shown in Table S1. Our proposed MoS_2_ grating/InSe flake photodetector not only showcases outstanding responsivity, detectivity, and ultrafast response speed down to the nanosecond scale but also outperforms traditional polarization-sensitive photodetector by demonstrating spectrally selective polarization sensitivity, significantly expanding its potential in optoelectronic applications.

## Conclusions

In summary, a spectrally selective polarization-sensitive near-infrared photodetector based on an all-vdW MoS_2_ grating/InSe flake heterostructure was proposed in this work. To enhance the light-matter interaction, multilayer MoS_2_ grating was integrated with an InSe flake, based on the advantages of inherent low optical loss and strong field confinement of TMDC compared to metals. The SPR mode excited by the MoS_2_ grating possesses a 229% higher Q-factor compared to that excited by the Ag grating, indicating lower loss and higher photoelectric conversion efficiency. In particular, the MoS_2_ grating supports both TM-excited plasmonic resonance at λ = 960 nm and TE-excited guided-mode resonance at λ = 790 nm, enabling spectrally selective polarization-sensitive photodetection in the NIR region due to its LD conversion behavior. Owing to the strong local field intensity induced by resonance, integrating an InSe flake with MoS_2_ gratings results in a significant 35-fold enhancement in photoluminescence. The proposed MoS_2_ grating/InSe flake photodetector exhibits a maximum responsivity and detectivity of 28.5 A/W and 9.81 × 10^12^ Jones for 960 nm laser, showing a 721- and 123-fold enhancement, respectively, compared to the pristine InSe device. Additionally, ultrafast operation speeds with a rise time of 195 ns and a decay time of 222 ns were also achieved, representing the fastest photoresponse speeds ever reported in InSe-based photodetectors. This study demonstrates the integration of 2D semiconductor gratings into plasmonic photodetectors, highlighting the advantages of TMDC MoS_2_ gratings over conventional metal gratings in enhancing the photoresponsivity and polarization selectivity. These findings underscore the potential of all-vdW heterostructures for future applications in metamaterials, plasmonics, and nanophotonics.

## Methods

### Device Fabrication

First, 330 nm of SiO_2_ was thermally grown on p^+^ doped silicon for insulation. The SiO_2_/p^+^-Si substrate was cleaned with acetone, isopropyl alcohol (IPA), and deionized (DI) water under ultrasonication for 5 min each, and then baked at 120 °C for 10 min to dry. Next, the photoresist (EPG-512) was spin-coated on the substrate at 1000 rpm for 11 s and 5000 rpm for 34 s, followed by a soft bake at 110 °C for 160 s. Digital Light Processing (DLP) maskless photolithography (UTA-IIIA, ARMS, Japan) was used to pattern the electrode with a 5 μm channel length, which was then developed with AD-10 developer for 40 s. Au/Cr (70/10 nm) was deposited using the e-gun evaporation process, and the lift-off process was performed using Remover PG at 60 °C for 2 h. The multilayer MoS_2_ and InSe flakes were prepared by mechanical exfoliation from high-quality bulk crystals (Purity >99.99%) and then transferred onto the as-prepared Au electrodes using the dry transfer technique to form a MoS_2_/InSe heterojunction. MoS_2_ gratings were fabricated by using e-beam lithography followed by a dry etching process. First, a PMMA (950 A4, Micro Chem.) resist was spin-coated onto the device at 1000 rpm for 90 s, yielding a ∼400 nm thick resist layer, followed by a soft bake at 180 °C for 3 min. A layer of conductive polymer (Espacer 300Z) was then spin-coated onto the PMMA layer at 2500 rpm for 90 s and baked at 80 °C for 3 min. The PMMA resist was patterned using e-beam lithography system (ELS-7800, Elionix) with a beam current of 200 pA and an acceleration voltage of 80 kV. The device was then developed in a solution of methyl isobutyl ketone (MIBK) and IPA at a 1:3 ratio for 1 min and rinsed in IPA for 1 min. The patterned PMMA was used as an etching mask. Subsequently, the exposed MoS_2_ was removed using inductively coupled plasma reactive-ion etching (Oxford Instrument/Plasmalab System 100 ICP-RIE) with C_4_F_8_ (50 sccm) and Ar (20 sccm) under 1000 W ICP power and 20 W RF power. The etching process was conducted for 1 min in 4 rounds to prevent resist hardening due to the temperature increase from extended etching time. Finally, the resist-mask was removed using Remover PG at 140 °C, which is higher than the reflow temperature of PMMA (125 °C), for 2 h, and then rinsed with acetone and IPA.

### Numerical Simulation

The absorption spectra and electromagnetic field distributions of the MoS_2_ grating structure were calculated by using the 3D model in COMSOL Multiphysics. A periodic boundary condition was set in *x*- and *y*-direction, and a perfect electric conductor boundary condition was set in the *z*-direction. The period, width, height, and length of the MoS_2_ grating were set to 650 nm, 325 nm, 136 nm, and 10 μm, respectively. Additionally, a top air medium with a thickness five times the period and a 100 nm bottom Au layer were designed. The real and imaginary parts of the refractive index (n, k) of MoS_2_ and Au were obtained from the RefractiveIndex.INFO Web site, based on reported works.^30,48^ The input source of the linearly polarized plane wave with varying polarization angles was set in the excitation port and monitored for reflection with the same port.

### Device Characterization

Raman and photoluminescence spectra were measured using micro-Raman spectroscopy (alpha300 RA, Raman-AFM Microscope, WITec) with an ultrahigh throughput spectrometer (UHTS 300, WITec). Scanning electron microscopy (FESEM-8010, HITACHI) was used for the morphological characterization and EDS mapping. The structure thickness and work function difference mapping were obtained by using tapping mode atomic force microscopy (AFM) and Kelvin probe force microscopy (KPFM) (Dimension ICON, Bruker).

### Photoresponse Measurement

The electrical properties of the MoS_2_ grating/InSe flake photodetector were measured by using a Keithley 2636A System SourceMeter combined with a probe station in an ambient environment. The light source was a supercontinuum white light laser (SuperK FIANIUM, NKT Photonics) combined with a monochromator (CS260B-Q-MC-D Monochromator, Newport) and a universal filter wheel (USFW-100, Newport), enabling the output of any single wavelength laser from 400 to 1700 nm. The output NIR light was passed through a half-wave plate and a linear polarizer to control its polarization orientation, and a neutral density (ND) filter was used to adjust the power of the light. Temporal photoresponse measurements were conducted using an acousto-optic modulator (AOM, Isomet M1210) to switch the light intensity on and off. The whole photodetection setup is shown in Figure S7.

## Supplementary Material



## Notes

OM images of MoS_2_ grating/InSe flake; AFM height profiles of MoS_2_ grating and InSe flake; EDX mapping and spectra of InSe flake; height-dependent absorption spectra of MoS_2_ grating; comparison of reflectance spectra for MoS_2_ and Ag gratings under TM and TE polarizations; polarization-dependent normalized photocurrent for planar MoS_2_/InSe device; comparison table of optoelectronic performance for various InSe-based photodetectors; schematic diagram of the measurement setup (PDF)
