# Supplementary material for: All-van-der-Waals Heterostructure of MoS2 Grating and InSe Flake for Spectrally Selective Polarization-Sensitive Photodetection in NIR Region
Source: ACS Nano. 2025 May 7;19(19):18545–55. doi: 10.1021/acsnano.5c02102 (PMC12096427; doi:10.1021/acsnano.5c02102)
Supplement: Supplementary file 1 [file nn5c02102_si_001.pdf]

Supporting Information

All-van-der-Waals heterostructure of MoS_2 grating and InSe flake for spectrally selective polarization-sensitive photodetection in NIR region

Yu-Te Chu,[†] *Po-Liang Chen,*[‡] *Shih-Hsiu Huang,*[§] *Shyam Narayan Singh Yadav,*[†] *Wei-Ren Syong,*[||] *Ching-Han Mao,*[†] *Yu-Jung Lu,*[||,#] *Chang-Hua Liu,*[‡] *Pin Chieh Wu,*[§,⊥,▽] *Ta-Jen Yen*[*,†]

[†] Department of Materials Science and Engineering, National Tsing Hua University, Hsinchu 30013, Taiwan

[‡] Institute of Photonics Technology, National Tsing Hua University, Hsinchu 30013, Taiwan

[§] Department of Photonics, National Cheng Kung University, Tainan 70101, Taiwan

[||] Research Center for Applied Sciences, Academia Sinica, Taipei 11529, Taiwan

[#] Department of Physics, National Taiwan University, Taipei 10617, Taiwan

[⊥] Center for Quantum Frontiers of Research & Technology (QFort), National Cheng Kung University, Tainan 70101, Taiwan

[∇] Meta-nanoPhotonics Center, National Cheng Kung University, Tainan 70101, Taiwan

* E-mail: tjyen@mx.nthu.edu.tw

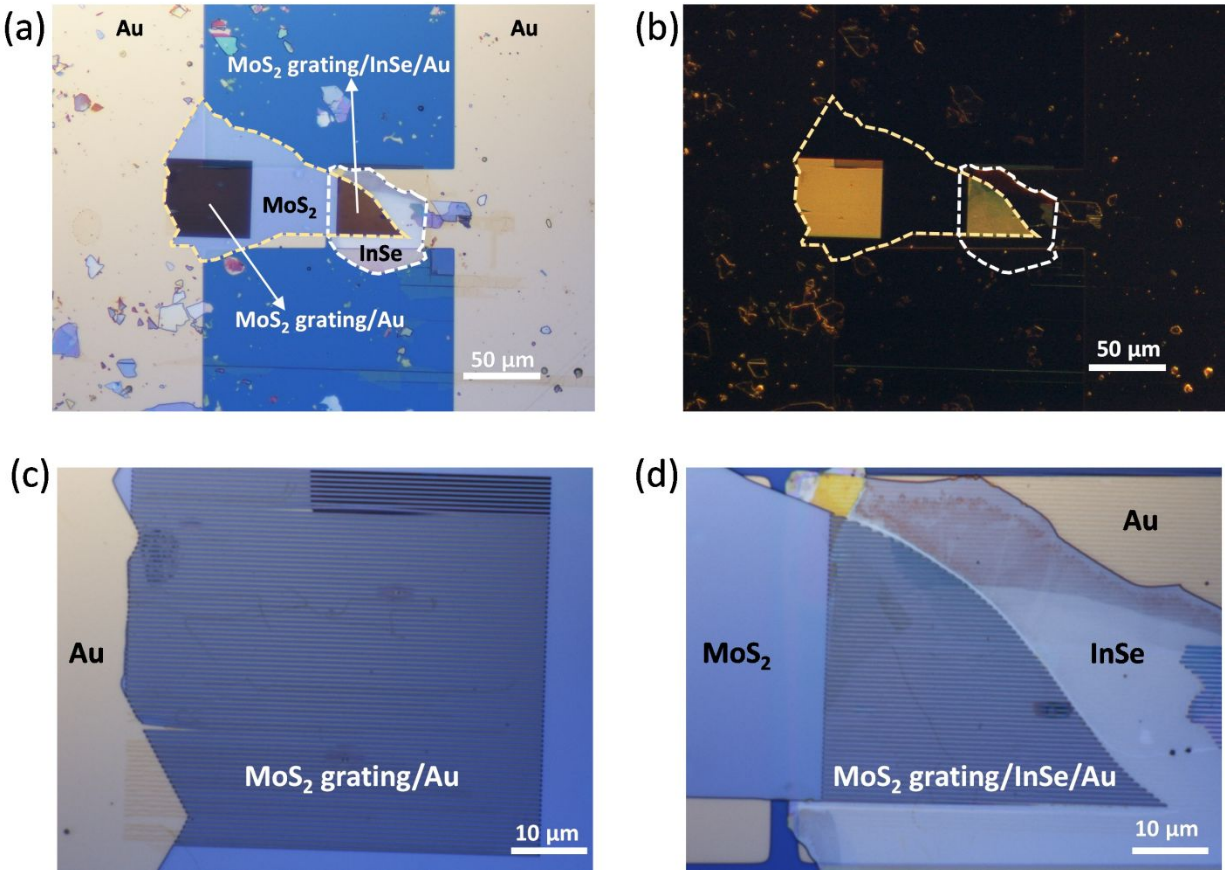

Figure S1. (a) Bright-field and (b) corresponding dark-field OM images of MoS_2 grating/InSe flake hybrid photodetector under low magnification. The regions of MoS_2 and InSe are defined by the yellow and white dashed lines, respectively. (c) OM images of the MoS_2 grating on Au electrode and (d) MoS_2 grating/InSe on Au electrodes under high magnification.

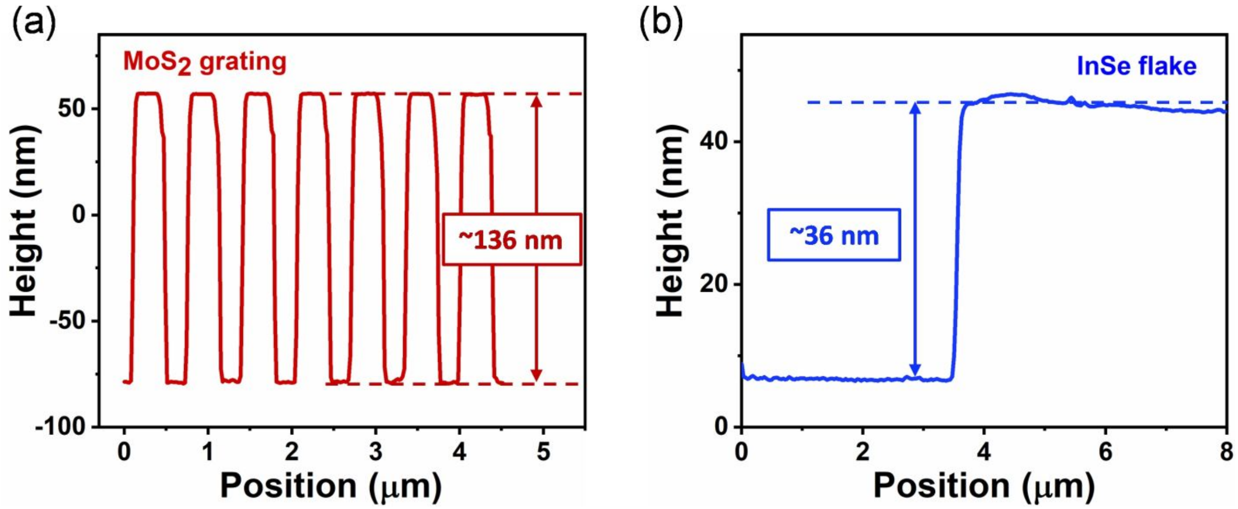

Figure S2. (a) AFM height profiles extracted from Figure 1(d) show that the height of MoS_2 grating is about 136 nm and (b) the thickness of InSe is about 36 nm.

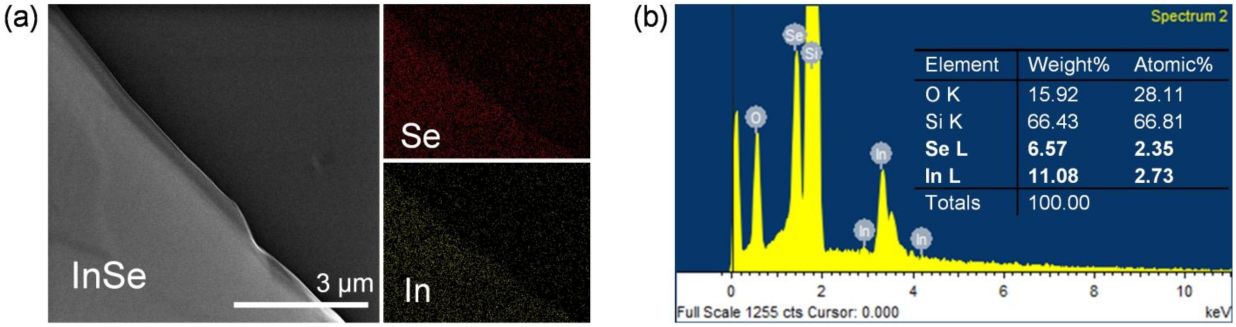

Figure S3. (a) SEM image of InSe flakes and EDX mapping of Se and In elements. (b) EDX spectra and atomic percentage of InSe flakes revealing an In-to-Se ratio of approximately 1:0.913.

Height-dependent absorption spectra of MoS_2 grating

To further explore the influence of grating height, the simulated absorption spectra with varying heights under the fixed period of P = 650 nm is shown in Figure S4a. The spectral positions of the magnetic resonance mode (cross symbol) exhibit a redshift as the grating height increases. However, such height-dependent properties make the control of its resonance wavelength challenging, owing to the inherently random thickness (i.e., grating height) of mechanically exfoliated MoS_2 flakes. By contrast, the third-order SPR modes (ring symbol) are observed to be insensitive to height, which relaxes the strict requirement for fabrication and enables the resonance wavelength to be designed solely by tuning the grating period. As a result, we selected the third-order SPR modes to design the resonance wavelength near the band edge of bulk InSe, with optimized geometrical parameters determined as P = 650 nm, W = 325 nm, and H = 136 nm.

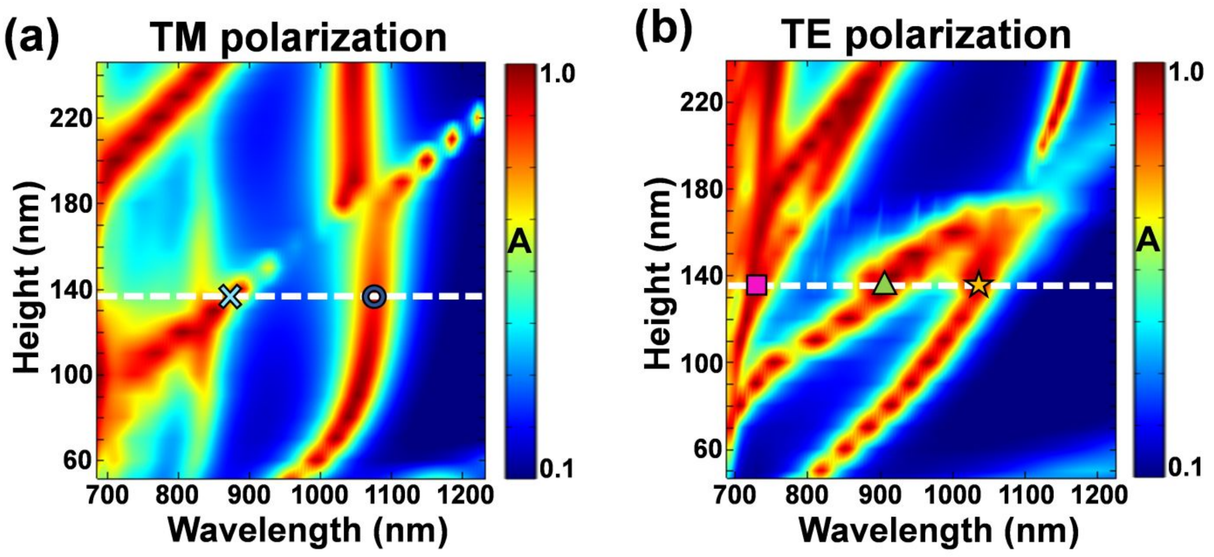

Figure S4. Color map of COMSOL simulated absorption spectra as a function of MoS_2 grating height under TM (a) and TE (b) polarizations.

Comparison of reflectance spectra for MoS_2 and Ag gratings under TM and TE polarizations

In Figure S5a, both MoS_2 grating and Ag grating exhibit a reflectance valley attributed to the SPR modes under TM polarization. Moreover, the resonance peak of the MoS_2 grating possesses a quality-factor (Q-factor) of 14.33, which is 229% higher than that of the Ag grating, indicating the inherent low-loss properties of the MoS_2 grating owing to its small imaginary parts of the permittivity. Furthermore, in Figure S5b, the MoS_2 grating displays multiple guided-mode resonances, whereas the Ag grating shows no resonance response under TE polarization in the NIR region.

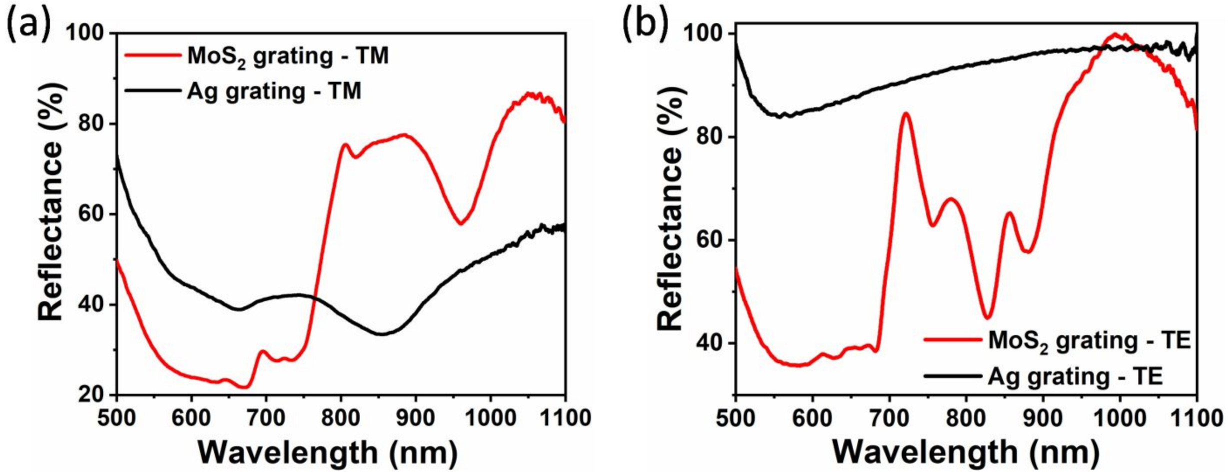

Figure S5. Comparison of the measured reflectance spectra for MoS_2 grating and Ag grating on Au substrate under (a) TM and (b) TE polarization.

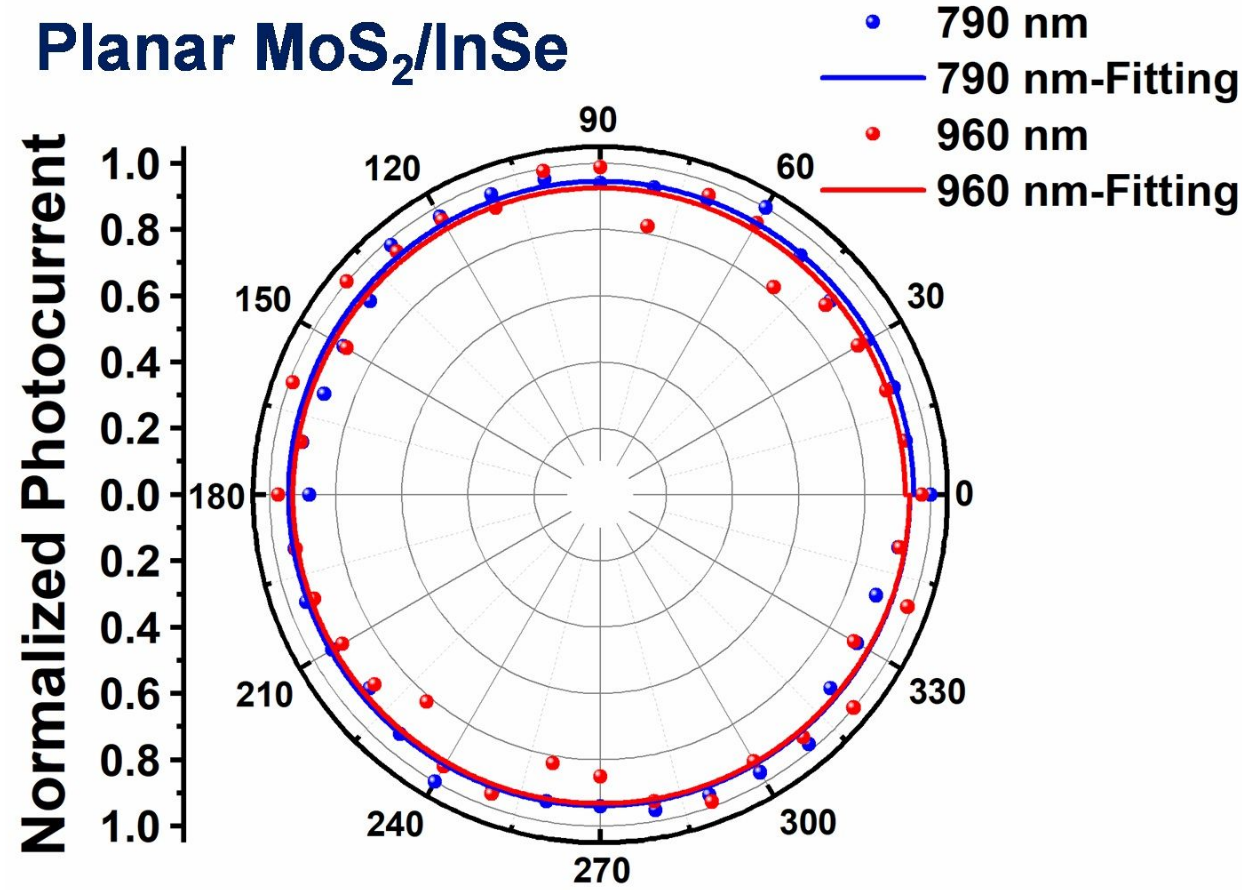

Figure S6. Polarization-dependent normalized photocurrent for planar MoS_2/InSe device at λ=790 nm and 960 nm.

Table S1. Comparison of the optoelectronic performance for various reported InSe-based NIR photodetectors. (All measured at $V_g = 0$ V)

Device	Bias (V)	λ (nm)	Responsivity (AW^{-1})	Detectivity (Jones)	Rise time/ Decay time	References
InSe/graphene electrode	10	1000	5.3	_	120/220 μs	[1]
γ-InSe	-5	785	7.47	4.56×10^{13}	27/27 μs	[2]
Surface-doped InSe	1	980	7870	1.48×10^{13}	1.65/4.8 s	[3]
p-n-InSe	0	980	0.0005	_	8.3/9.6 ms	[4]
InSe/$PdSe_2$	1	1650	58.8	1×10^{10}	160/180 ms	[5]
SnSe/InSe	0	808	0.35	5.8×10^{10}	260/170 ms	[6]
BP/InSe	1	1550	43.11	_	22/48 ms	[7]
VP/InSe	1	1064	182.8	7.86×10^{12}	17/18 μs	[8]
MXene grating/InSe	3	785	3000	1×10^{11}	0.5/26 ms	[9]
Triangular Au nanoparticle/InSe	0	685	0.244	3.35×10^{12}	23/25 ms	[10]
MoS_2 grating/InSe	-2	960	28.5	9.81×10^{12}	195/222 ns	This work

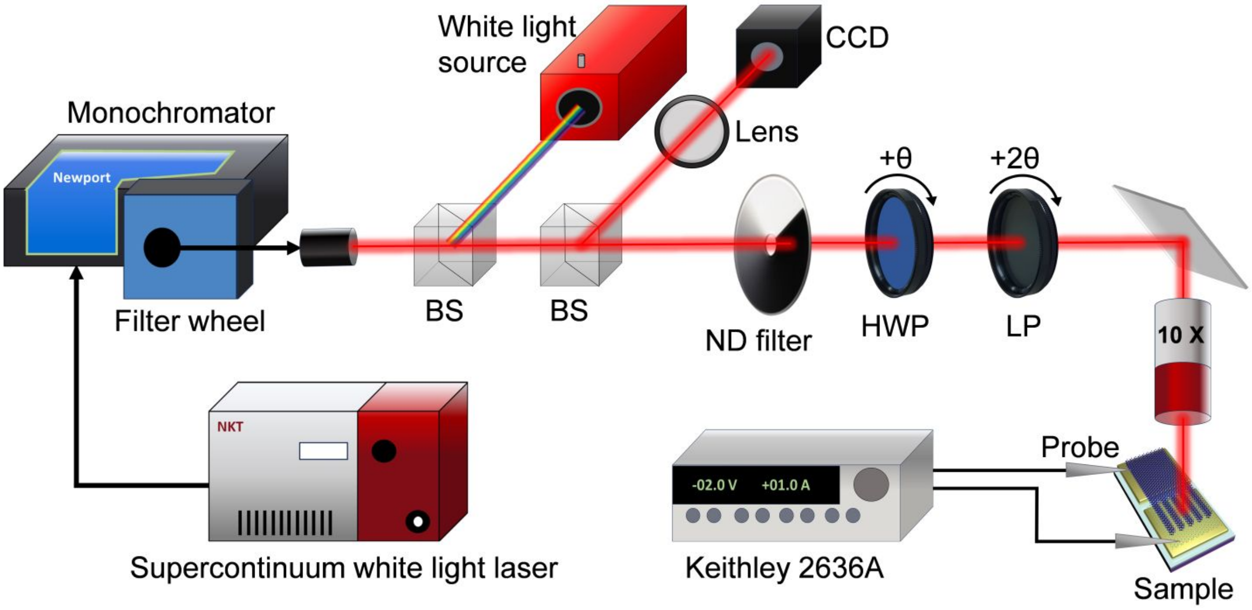

Figure S7. Schematic diagram of the measurement setup for the MoS_2 grating/InSe flake spectrally selective polarization-sensitive photodetector, where BS is a beam splitter, HWP is a half-wave plate, and LP is a linear polarizer.

REFERENCES

(1) Luo, W.; Cao, Y.; Hu, P.; Cai, K.; Feng, Q.; Yan, F.; Yan, T.; Zhang, X.; Wang, K. Gate tuning of high-performance InSe-based photodetectors using graphene electrodes. *Advanced Optical Materials* **2015**, *3* (10), 1418-1423.
(2) Wang, X.; Wen, B.; Gao, S.; Li, X.; Lin, Z.; Du, L.; Zhang, X. Strong Anisotropy of Multilayer γ-InSe-Enabled Polarization Division Multiplexing Photodetection. *Advanced Photonics Research* **2022**, *3* (12), 2200119.
(3) Jang, H.; Seok, Y.; Choi, Y.; Cho, S. H.; Watanabe, K.; Taniguchi, T.; Lee, K. High-performance near-infrared photodetectors based on surface-doped InSe. *Advanced Functional Materials* **2021**, *31* (3), 2006788.
(4) Patil, C.; Dong, C.; Wang, H.; Nouri, B. M.; Krylyuk, S.; Zhang, H.; Davydov, A. V.; Dalir, H.; Sorger, V. J. Self-driven highly responsive pn junction InSe heterostructure near-infrared light detector. *Photonics Research* **2022**, *10* (7), A97-A105.
(5) Ahmad, W.; Liu, J.; Jiang, J.; Hao, Q.; Wu, D.; Ke, Y.; Gan, H.; Laxmi, V.; Ouyang, Z.; Ouyang, F. Strong interlayer transition in few-layer InSe/PdSe2 van der Waals heterostructure for near-infrared photodetection. *Advanced Functional Materials* **2021**, *31* (43), 2104143.
(6) Yan, Y.; Abbas, G.; Li, F.; Li, Y.; Zheng, B.; Wang, H.; Liu, F. Self-Driven High Performance Broadband Photodetector Based on SnSe/InSe van der Waals Heterojunction. *Advanced Materials Interfaces* **2022**, *9* (12), 2102068.
(7) Cao, R.; Wang, H. D.; Guo, Z. N.; Sang, D. K.; Zhang, L. Y.; Xiao, Q. L.; Zhang, Y. P.; Fan, D. Y.; Li, J. Q.; Zhang, H. Black phosphorous/indium selenide photoconductive detector for visible and near-infrared light with high sensitivity. *Advanced Optical Materials* **2019**, *7* (12), 1900020.
(8) Ahmad, W.; Rehman, M. U.; Pan, L.; Li, W.; Yi, J.; Wu, D.; Lin, X.; Mu, H.; Lin, S.; Zhang, J. Ultrasensitive Near-Infrared Polarization Photodetectors with Violet Phosphorus/InSe van der Waals Heterostructures. *ACS Applied Materials & Interfaces* **2024**.
(9) Yang, Y.; Jeon, J.; Park, J.-H.; Jeong, M. S.; Lee, B. H.; Hwang, E.; Lee, S. Plasmonic transition metal carbide electrodes for high-performance InSe photodetectors. *ACS nano* **2019**, *13* (8), 8804-8810.
(10) Dai, M.; Chen, H.; Feng, R.; Feng, W.; Hu, Y.; Yang, H.; Liu, G.; Chen, X.; Zhang, J.; Xu, C.-Y. A dual-band multilayer InSe self-powered photodetector with high performance induced by surface plasmon resonance and asymmetric Schottky junction. *ACS nano* **2018**, *12* (8), 8739-8747.
